# Direct amplification of Bordetella pertussis DNA purified from nasopharyngeal swabs by a low-cost, fast (60-second), and equipment-free method

**DOI:** 10.17843/rpmesp.2022.393.10865

**Published:** 2022-09-30

**Authors:** Eduardo Juscamayta-López, Faviola Valdivia, María Pía Soto, Helen Horna, Brenda Nureña

**Affiliations:** 1 Laboratorio de Infecciones Respiratorias Agudas, Centro Nacional de Salud Pública, Instituto Nacional de Salud, Lima, Peru. Laboratorio de Infecciones Respiratorias Agudas Centro Nacional de Salud Pública Instituto Nacional de Salud Lima Peru

**Keywords:** Point-of-Care Testing, Isolation & Purification, DNA, Bordetella pertussis, Cellulose, Molecular Diagnostic Techniques, Real-Time Polymerase Chain Reaction, LAMP loop-mediated isothermal amplification, Whooping Cough

## Abstract

**Objective.:**

To develop and evaluate a low-cost cellulose-based method for rapid purification and direct amplification of Bordetella pertussis DNA from nasopharyngeal swabs.

**Materials and methods.:**

We prepared cellulose discs and evaluated different parameters (lysis/wash buffers, number of discs and DNA elution). The method was coupled to a direct real-time PCR (qPCR) amplification and the performance was estimated using nasopharyngeal swabs that were positive (n=100) and negative (n=50) for B. pertussis DNA by qPCR, compared to the silica column-based method. We calculated sensitivity, specificity, positive predictive value (PPV) and negative predictive value (NPV) and the degree of agreement. The feasibility of the rapid method to be coupled to a loop-mediated isothermal amplification colorimetric assay (LAMP) was evaluated.

**Results.:**

The rapid method, with a cellulose disk and lysis and wash buffer containing PVP-40 and Tween 20, respectively, showed a greater capacity to purify amplifiable DNA from B. pertussis. The method had a sensitivity of 89.0% (95%CI: 80.2%-94.9%) and a specificity of 98.5% (95%CI: 92.1%-100.0%), with a good degree of agreement (Kappa=0.867; 95%CI: 0.788 - 0.946), compared to the reference method. The PPV and NPV were 98.6% (95%CI: 92.7.2%-100.0%) and 88.2% (95%CI: 78.7%-94.4%), respectively. Successful amplification by LAMP was evident, and comparable results were obtained with the silica column method.

**Conclusion.:**

The developed method is simple, low-cost and equipment-free for rapid (60 seconds) DNA collection at the point of care, and can be implemented in various molecular techniques aimed at the timely diagnosis and epidemiological study of pertussis.

## INTRODUCTION

Whooping cough or pertussis is a highly transmissible respiratory infection caused by the bacterium *Bordetella pertussis*; it is a disease of great importance for public health worldwide due to how severe it can be; it especially affects children under five years of age, mostly neonates ^1^.

In Peru, pertussis is considered an endemic disease with peaks of infection every 3 to 5 years ^2^, therefore a rapid and highly sensitive diagnostic method is key to controlling the disease and limiting its transmission. Molecular diagnosis of pertussis is based on real-time polymerase chain reaction (qPCR) using DNA obtained from nasopharyngeal swabs ^3^. However, the purification of nucleic acids from clinical samples is a complex task that demands time, specialized personnel, and resources, which makes this procedure a bottleneck that delays the diagnosis of the disease ^4^.

The constant search for new methods of nucleic acid purification currently proposes a range of possibilities; solid-phase extraction kits are the most widely used for subsequent molecular applications ^5^. Most of these commercial kits are based on columns with silica membranes and are based on the interaction of nucleic acid charges and the matrix, under strict alkaline conditions, thus generating a selective binding of nucleic acids ^6^ and later obtaining the genetic material; however, the process demands time due to its numerous stages, as well as the use of equipment and trained personnel ^7^. Magnetic bead technology has become a widely used purification process in recent decades, in which, by complementary hybridization, nucleic acids are attached to magnetic particles, which are subsequently immobilized, allowing the removal of contaminants ^8^. Although this method is relatively fast (15 to 30 min) and does not require centrifugation, its application outside the laboratory is challenging ^9^. In addition, residues of magnetic beads, or their direct use in PCR, can inhibit the amplification process leading to lower sensitivity or false negatives ^10^.

Other membrane-based nucleic acid extraction methods, such as Flinders Technology Associates (FTA) cards and Fusion 5 silica filters, have shown promising results in different biological samples ^11^ ^-^ ^13^. The advantage of these methods lies in the elimination of the elution step of the genetic material and the direct amplification of nucleic acids from membranes ^13^. However, they require multiple steps in the process and the use of equipment, so they are discarded as simple and fast methods ^14^.

Other cellulose-based techniques have proven useful in obtaining nucleic acids from different organisms, including plant ^15^ and animal ^16^ tissues, as well as cell lines ^17^. This reserch aimed to develop and evaluate a low-cost cellulose disk-based method for rapid (60 s) purification and direct amplification of *Bordetella pertussis* DNA from nasopharyngeal swabs.

**KEY MESSAGES**

Motivation for the study: whooping cough continues to be an endemic disease that affects infants more severely, therefore molecular tools are needed to reduce the time and cost of obtaining *B. pertussis* DNA for rapid detection and containment of transmissibility.

Main findings: the rapid DNA extraction method showed a good level of concordance with the purification by silica columns, obtaining DNA ready for direct amplification in 60 seconds.

Implications: the developed method is simple, low-cost, and equipment-free for obtaining DNA, which will allow timely diagnosis and molecular surveillance of whooping cough in places with limited resources.

## MATERIALS AND METHODS

### Study design and sample collection

This is a retrospective study that aimed to develop and evaluate a cellulose disc-based method for rapid purification and direct amplification of *B. pertussis* DNA using nasopharyngeal swabs obtained from pertussis cases confirmed by real-time PCR by the National Reference Laboratory for Acute Respiratory Infections of the Instituto Nacional de Salud (LRNIRA-INS), during the period from 2018 to 2019.

### Preparation of cellulose disks

Cellulose disks of 6 mm diameter were prepared from Whatman 2 paper (GE Healthcare) using a punch, which were subsequently sterilized by dry heat and stored at room temperature until use.

### Evaluation of lysis and wash buffers

In order to identify the best buffers, we evaluated two lysis and wash solutions (Table 1) using clinical specimens positive for *B. pertussis* DNA by qPCR and with Ct (Cycle Threshold) values of 24.71 and 24.47, respectively. We carried out serial dilutions in duplicate with phosphate buffer saline (PBS) up to 10^-5^ for each clinical specimen.Briefly, the methodology consisted of mixing 100 μL of sample in 200 μL of lysis buffer, gently shaking the lysate and placing the cellulose disk for 15 s in the solution, then transferring the disk to 200 μL of wash buffer for 1 min, and finally transferring the disk to the amplification mixture.

**Table 1:** Composition of lysis and wash buffers.

*Buffer*	Composition	Reference
LB#1	20 mM Tris [pH8], 25 mM NaCl, 2.5 mM EDTA, 0.05% SDS, 2% PVP-40	Mason and Botella, 2020
LB#2	20 mM Tris [pH8], 25 mM NaCl, 2.5 mM EDTA, 0.05% SDS	Mason and Botella, 2019
WB#1	10 mM Tris [pH8], 0.1% Tween 20	Zou *et al.*, 2017
WB#2	10 mM Tris [pH8]	Mason and Botella, 2019

LB: lysis buffer. WB: wasH buffer. NaCl: sodium chloride. EDTA: ethylenediaminetetraacetic acid. SDS: sodium dodecylsulfate. PVP-40: polyvinylpyrrolidone.

In order to evaluate the loss of genetic material contained in the cellulose disk by prolonged incubation time in the wash buffer, 100 μL of sample was added to 200 μL of lysis buffer 1 (LB#1), the solution was vortexed for a few seconds and the cellulose disk was placed for 1 min, with a light manual agitation; then the disk was transferred to 200 μL of wash buffer 1 (WB#1) where it remained for 18 h at 4 °C.

### Evaluation of the number of cellulose discs

We decided to evaluate whether the use of more than one cellulose disc could improve DNA collection and detection sensitivity of the rapid purification method. For this purpose, a panel of dilutions was created from a nasopharyngeal swab sample positive for *B. pertussis* DNA with Ct=21.71. The sample was diluted in PBS in duplicate to 10^-5^. Purification of the genetic material from each dilution was obtained according to the aforementioned procedure, including one or two cellulose disks per sample, as appropriate. DNA obtained by the rapid purification method, in both cases, was analyzed by real-time PCR based on the 481-insertion sequence (IS*481*).

### Elution of DNA captured on the cellulose disk

The DNA elution step was evaluated using (1) Tris-ethylenediaminetetraacetic acid (TE: 10 mM Tris, pH 8.5, 0.1 mM EDTA) and (2) dNTPs solution (400µM). The process was carried out in duplicate, mixing 100 μL of sample in 200 μL of LB#1, the solution was vortexed for a few seconds and the cellulose disk was placed for 1 min with a light manual agitation, then the disk was transferred to 200 μL of WB#1 for 1 min. After the washing step, one disk was transferred to the amplification mixture and the other, divided in two, to 50 μL of each elution solution and incubated for 5 min at room temperature. In a subsequent assay, the effect of a heat treatment was evaluated, for which two conditions were tested: incubation of the disc in elution solution at room temperature and with heating at 80 °C, both for 5 min.

### Purification of nucleic acids using commercial kit based on silica columns

We used the PureLink Genomic DNA Mini Kit (Invitrogen) purification kit for DNA purification from nasopharyngeal swab samples, following the manufacturer’s specifications. Briefly, 200 μL of sample was used, following the steps of digestion with proteinase K at 55 °C for 1 h, lysis and washing with their respective buffers. DNA was obtained in 100 μL of elution buffer and stored at 4 °C for subsequent qPCR analysis.

### Rapid cellulose disk-based nucleic acid purification and direct amplification

**Figure 1:**
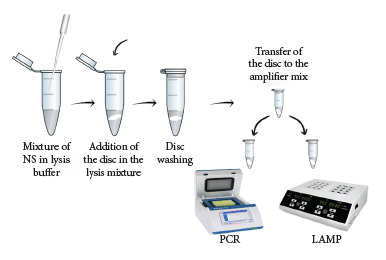
Flowchart of the rapid purification method coupled to direct amplification of *B. pertussis *DNA. Total DNA obtained by a simple and rapid (60 s) cellulose disk-based method available for qPCR or LAMP molecular analysis.

The optimized extraction method consisted of mixing 100 μL of nasopharyngeal swab sample with 200 μL of LB#1. The mixture was vortexed for 15 s and the cellulose disk was added. Then, with the help of a sterile 1000 μL tip, the disc was transferred to 200 μL of WB#1, gently vortexed for a few seconds and left to incubate at room temperature for 1 min. Finally, the disc was placed in the previously prepared amplification mixture (Figure 1).

### Direct amplification by real-time PCR and LAMP

To compare the performance of the new rapid method with the conventional DNA extraction method, we used 150 clinical samples that were positive (n=100) and negative (n=50) for *B. pertussis* DNA by qPCR. Genomic DNA from these samples was extracted using a commercial kit and the cellulose disk-based method in parallel and evaluated by real-time PCR. Real-time PCR assays were carried out according to the methodology described by Tatti *et al*. (2011), consisting of a multiplex assay for detection of IS*481*, pIS*1001* and hIS*1001* and the RNase P gene as an internal control. Amplification was carried out on the Rotor-Gene Q thermal cycler (Qiagen) using the following cycling parameters: 50 °C for 2 min, enzyme activation at 95 °C for 10 min and 45 cycles of amplification at 95 °C for 15 s and 60 °C for 1 min.

LAMP assays were carried out with Loopamp D RNA/DNA amplification reagent (Eiken Chemical Co., Ltd., Tokyo, Japan) and primers targeting the MaoC region were used (Juscamayta et al., unpublished data). The LAMP reaction consisted of a total volume of 25 μL with 40 pmol of internal primers (FIP and BIP), 5 pmol of external primers (F3 and B3), 20 pmol of loop-forming primers (LF and LB) and the cellulose disk. The tubes were incubated at 65 °C for 60 min and at 80 °C for 5 min to inactivate the reaction. The results were visually analyzed, a positive sample being determined by the brown to green color change in the reaction solution.

### Statistical analysis

R v4.0.5 and Stata/MP v15.0 were used to conduct the statistical analysis. Ct values were compared by one-way analysis of variance (ANOVA), after assessment of normality using the Shapiro-Wilk test. Positive and negative DNA real-time PCR results obtained by the rapid purification method and based on silica columns were analyzed in a 2x2 contingency table. Kappa concordance coefficients were used during the analysis ^18^. Ninety-five percent confidence intervals (CI) were calculated, and bilateral p<0.05 was considered significant for all statistical analyses.

### Ethical aspects

This study was carried out under the research protocol approved by the Institutional Research Ethics Committee of the INS (code: OI-032-18).

## RESULTS

### Evaluation of lysis and wash buffers and number of cellulose disks

**Figure 2:**
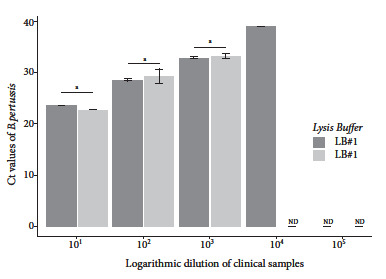
Real-time PCR Ct values of B. pertussis DNA purified with different lysis solutions. DNA was obtained from dilutions of nasopharyngeal swabs from individuals who tested positive for B. pertussis DNA by qPCR.

**Figure 3:**
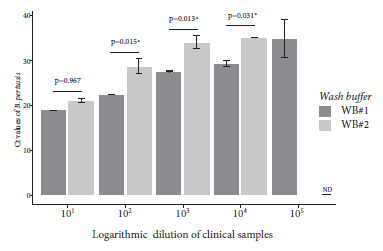
Real-time PCR Ct values of *B. pertussis *DNA purified with different washing solutions. DNA was obtained from dilutions of nasopharyngeal swabs from individuals who tested positive for *B. pertussis *DNA by qPCR.

Evaluation of the lysis and wash buffers resulted in lower Ct values for the combination of LB#1 based on PVP-40 and WB#1 containing Tween 20. The rapid DNA extraction method using LB#1 and LB#2 was able to purify *B. pertussis* DNA from a diluted clinical sample to 10^-4^ and 10^-3^, respectively; while the wash buffer was able to purify *B. pertussis* DNA to 10^-5^ and 10^-4^ using WB#1 and WB#2, respectively. Although slight differences were found between the Ct values obtained with the lysis buffers for dilutions 10^-1^ through 10^-3^, these differences were not statistically significant (p>0.05) (Figure 2). In contrast, the difference between the Cts achieved with the wash buffers was statistically significant for dilutions between 10^-2^ and 10^-4^ (Figure 3).

The exposure of the cellulose disc for a prolonged time (18 h) in washing buffer did not affect the detection of *B. pertussis* DNA by real-time PCR, and no significant differences were found between the Cts values for IS481 and RNase P obtained after incubation of the disc for 1 min (Ct_*IS481*_=16.42-20.99 and Ct_*RnasaP*_=25.31-30.84) and exposure for 18 h (Ct_*IS481*_=17.21-21.96 and Ct_*RnasaP*_=27.11-32.89). Also, working with two cellulose discs per clinical sample, the rapid method was able to detect *B. pertussis* DNA up to a dilution of 10^-2^ (Ct=15.46-26.34), and while using one disc it was able to detect *B. pertussis* DNA up to dilution 10^-4^ (Ct= 4.77-35.62) (Figure S1).

### Elution of DNA captured on the cellulose disk

In order to verify the presence of DNA on the cellulose disk, we carried out rapid purification assays using elution solutions including TE buffer and dNTPs solution, which were compared to the rapid purification method and direct amplification without elution. Both solutions were able to elute *B. pertussis* DNA from the cellulose disk, resulting in similar Cts values for IS*481*, with a median of 23.94 and 26.56 for TE and dNTPs, respectively (Table S1). The rapid purification method coupled to direct amplification without elution resulted in lower Cts values than those obtained with TE and dNTPs, for both the internal control gene and the *B. pertussis*-specific target (Table S1). After adding temperature treatment to the elution step, we found no significant differences between the Cts values for IS*481* and *RNase P* from each evaluated elution buffer (Table S2).

### Evaluation of cellulose-based rapid DNA purification method

**Figure 4:**
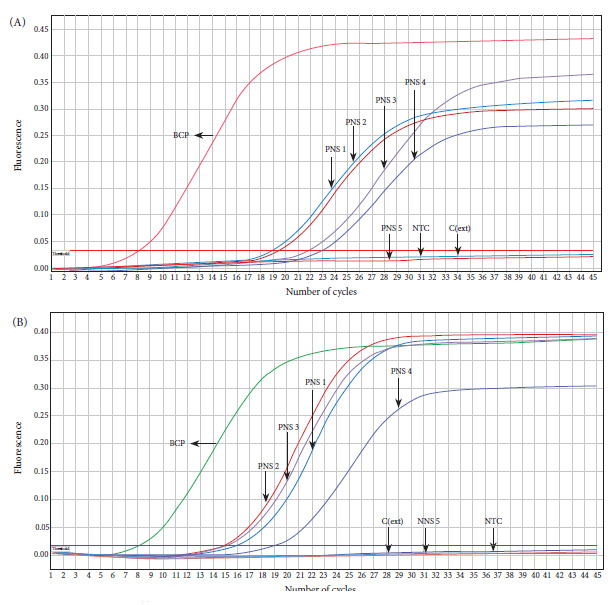
Amplification curves for IS481 of *B. pertussis *DNA from nasopharyngeal swab samples purified by the rapid (A) and conventional (B) methods.

The sensitivity, specificity, positive predictive value, and negative predictive value of the rapid methodology for direct DNA purification and amplification of *B. pertussis* resulted in 89.0% (95%CI, 80.2%-94.9%), 98.5% (95%CI, 92.1%-100.0%), 98.6% (95%CI, 92.7%-100.0%) and 88.2% (95%CI, 78.7%-94.4%), respectively, compared with the silica column-based extraction and qPCR amplification method (reference methodology) (Figure 4, Table S3). The conventional extraction method was able to detect *B. pertussis* DNA in 82% of the positive clinical specimens (82/100), whereas the rapid method, *B. pertussis* DNA was detected in 74% of the positive specimens (74/100). Of the 68 samples negative for *B. pertussis* DNA obtained by the conventional method, 67 were also negative by the rapid method, with 1 false positive. Likewise, of the 82 positive samples for *B. pertussis* DNA purified by the conventional method, 73 were positive by the rapid cellulose-based method, with 9 false negatives. The degree of agreement between the results of both methods resulted in a kappa coefficient of 0.867 (95%CI 0.788 - 0.946).

### Direct amplification using LAMP isothermal methodology

**Figure 5:**
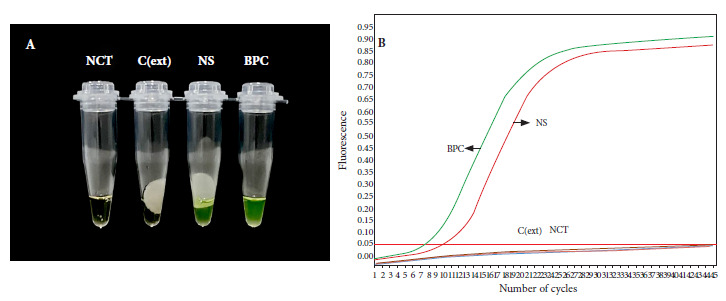
Direct LAMP amplification of B. pertussis DNA purified by the rapid cellulose-based method. Reactions were considered positive for LAMP product if they changed color from brown to green (A). DNA was obtained from a nasopharyngeal swab sample by the rapid and conventional method and analyzed by LAMP (A) and real-time PCR (B), respectively.

In order to evaluate the feasibility of the rapid purification method coupled to direct amplification with a rapid and low-cost molecular method, we carried out a loop-mediated isothermal amplification (LAMP) assay using a clinical specimen positive for *B. pertussis* DNA by qPCR. DNA was again purified from the clinical specimen by the rapid and conventional method, followed by amplification by LAMP and real-time PCR, respectively. The LAMP assay was able to specifically detect *B. pertussis* DNA obtained by the rapid method that was visualized through color change from brown to green (Figure 5A). A similar result was observed in the real-time PCR assay, which was able to detect *Bordetella pertussis* DNA (Ct=12.79) purified by the conventional method in the same sample (Figure 5B). No amplification was evident in the negative controls in any of the assays (Figure 5).

## DISCUSSION

Rapid and simple molecular methods are necessary to enhance diagnosis and control the spread of highly contagious infectious diseases such as whooping cough. However, the main difficulty of molecular detection is that it requires the purification of nucleic acids which demands time and the use of expensive reagents and equipment ^19^ that, in addition, may be in short supply during periods of outbreaks, as demonstrated by the COVID-19 pandemic. In this study we demonstrate the direct amplification of purified *B. pertussis* DNA from nasopharyngeal swabs by a low-cost, rapid (60 s) method without the use of sophisticated equipment.

Several papers have evaluated different components of buffers for extraction of nucleic acids from bacteria, parasites, fungi, and viruses ^15^ ^-^ ^17^ ^,^ ^20^. In this study, we chose lysis and wash buffers based on the ability to purify nucleic acids from diluted nasopharyngeal swab specimens. The lysis buffer containing PVP-40 was able to purify *B. pertussis* DNA at a higher dilution that what was detected by qPCR (Figure 2). PVP-40 is a nontoxic chemical agent that helps to minimize the effect caused by interfering compounds on DNA amplification ^21^. Moreover, it has been shown to be effective in different types of samples ^17^. On the other hand, the Tween 20-based wash buffer showed the highest analytical sensitivity (Figure 3), contrary to the study of Mason and Botella ^15^, who reported negative interference of this compound in amplification reactions.

The rapid method using only one cellulose disk was able to purify amplifiable *B. pertussis* DNA from a clinical sample twice as diluted as using two cellulose disks (Figure S1). Similar results were reported by Zou *et al*. who were able to detect *Actinobacillus pleuropneumoniae* DNA purified by cellulose disk and showed that the amount and yield of purified DNA decreases with increasing number of disks, probably due to higher adsorption of PCR components on cellulose membranes ^17^.

Elution of the genetic material in separate solutions was not reflected in a better yield during amplification, on the contrary, direct release of the cellulose disc into the amplification mixture resulted in lower Ct values (Table S1), suggesting a higher amount of recovered nucleic acids, where the influencing factor was probably the dNTPs, which play an important role in the desorption of DNA from different matrices, as demonstrated by Tanaka *et al*, who compared the recovery yield of DNA extracted with aminosilane-modified magnetic nanoparticles, concluding that the use of dNTPs results in very efficient elution, as dNTPs are integral components of PCR amplification ^22^. Previous studies have shown that heat treatment leads to higher DNA elution efficiency, resulting in better detection by qPCR ^23^, however, our results are contrary to these, as no significant differences were observed with temperature treatment (Table S2), so we decided to omit the elution step, obtaining a simple and fast methodology composed of only three steps (Figure 1).

DNA purified by cellulose disk showed an almost perfect degree of concordance (kappa=0.867; 95%CI 0.788 - 0.946) with the results obtained by the silica column-based method (Figure 4), according to the classification by Landis and Koch ^24^. Of the 100 evaluated positive clinical samples, 18 were negative for *B. pertussis* DNA using the silica column-based methodology, whereas the rapid method yielded 26 negative samples, of which 17/26 matched the results of the conventional method. The relatively high number of false negatives could be due to the integrity of the genetic material being affected by the freeze-thaw cycles of the stored clinical samples, resulting in bias regarding the amplification with an increase in false negatives during the molecular detection of the pathogen ^25^.

The rapid methodology resulted in a sensitivity and specificity of 89.0% (95% CI, 80.2%-94.9%) and 98.5% (95% CI, 92.1%-100.0%), respectively (Table S3). A study of recombinase amplification using boiling-purified *B. pertussis* DNA showed a sensitivity of 85% ^26^; however, as this is a heat-based technique only, some PCR inhibitory components are not removed from the samples, negatively affecting the amplification reaction ^27^. Additionally, purification by boiling requires about 15 min, whereas our rapid cellulose method requires 60 s. Xu *et al*. proposed a purification method based on modified magnetic nanoparticles (MNP), which showed amplification results concordant with those obtained by a commercial kit in human blood samples ^28^. Although the MNP methodology has shown good performance in obtaining DNA in an average time of 10 min, it requires the use of equipment and relatively complex preparation of nanoparticles. Centrifugation-free devices have been developed for rapid DNA extraction in 2 min with performance similar to that purified by a commercial kit ^29^; however, they are not coupled to direct amplification by PCR or LAMP and are limited to the analysis of a single sample. To date, there are no reports of rapid methods for direct purification and amplification of *B. pertussis* DNA from nasopharyngeal swabs; therefore, a new simple and rapid alternative for purification of nucleic acids in this type of clinical samples is proposed by our study.

In this study, in addition, the rapid purification method was coupled to direct amplification by loop-mediated isothermal amplification (LAMP); this is a sensitive, rapid, and inexpensive molecular technique that has been widely used in the detection of respiratory pathogens, including influenza ^30^, *B. pertussis* ^31^, and SARS-CoV-2 ^32^. Our assay evidenced successful amplification by LAMP, obtaining results comparable to the silica column method. Similar results were obtained by Aula *et al*. who, using cellulose strips, achieved successful LAMP amplification up to a dilution of 2^-13^ of a sample containing Schistosoma eggs, demonstrating the high sensitivity of the rapid purification method coupled to LAMP amplification ^20^. Similarly, Kellner *et al*. achieved positive results for the detection of SARS-CoV-2 by LAMP amplification, where the sensitivity of the cellulose strip purification was identical to the magnetic bead method ^33^.

Our cellulose disk-based nucleic acid purification method has the following advantages: 1) it is a rapid methodology that allows DNA purification in 60 s from nasopharyngeal swabs; 2) it is low cost and does not require complex equipment; 3) it is a simple method (three steps) that generates genetic material in a very short time to be used in various molecular techniques aimed at the diagnosis and epidemiological study of whooping cough (Figure 1); and 4) it has the potential to purify not only DNA, but also RNA, and be scaled up for the molecular detection of any pathogen, including SARS-CoV-2. These features make the method a very useful alternative for rapid purification and molecular detection of *B. pertussis* DNA in environments with minimal equipment conditions, reducing the need for transportation and storage of biological samples.

The main limitation of this study is the retrospective use of clinical samples, which could affect the integrity of the genetic material and consequently impact the performance of the rapid DNA purification method. Unlike the silica column-based purification method, the cellulose-based method does not concentrate the nucleic acids in the sample. However, the advantage of the cellulose disk is that it absorbs the genetic material from small sample volumes and allows rapid purification ^15^.

In conclusion, this study demonstrates that the cellulose disk-based method allows rapid purification (60 s) and simultaneous molecular detection of *B. pertussis* DNA by qPCR or LAMP from nasopharyngeal swabs, with performance very similar to the conventional methodology based on silica columns. The rapid method could be very useful in strengthening timely diagnosis and molecular surveillance of whooping cough at the first level of care to help control and prevent the disease.
