# Supplementary material for: Direct amplification of Bordetella pertussis DNA purified from nasopharyngeal swabs by a low-cost, fast (60-second), and equipment-free method
Source: Rev Peru Med Exp Salud Publica. 2022 Sep 30;39(3):312–20. doi: 10.17843/rpmesp.2022.393.10865 (PMC11397763; doi:10.17843/rpmesp.2022.393.10865)
Supplement: Supplementary files: — supplementary material. [file rpmesp-39-03-10865-s001.docx]

**Material suplementario**

**Tabla S1.** Elución del ADN capturado en el disco de celulosa en diferentes soluciones

| **Muestra HN** | **Método de elución de ADN** | | | | | |
| --- | --- | --- | --- | --- | --- | --- |
|  | **AD** | | **TE** | | **dNTPs** | |
|  | Ct  *RNasa P* | *Ct* IS*481* | Ct  *RNasa P* | *Ct* IS*481* | Ct  *RNasa P* | Ct IS*481* |
| 1 | * | * | 25,11 | 28,93 | 32,73 | 36,54 |
| 2 | * | * | 23,49 | 21,94 | 32,18 | 28,00 |
| 3 | 23,38 | 19,56 | 29,12 | 25,93 | 29,33 | 25,12 |
| 4 | 25,31 | 16,42 | 30,29 | 20,80 | 30,84 | 20,64 |

**HN:** hisopado nasofaríngeo. **AD:** amplificación directa sin elución. **TE:** tris-ácido etilendiaminotetraacético. **dNTPs:** deoxinucleósido trifosfato. **Ct:** *Cycle Threshold*. *****muestras no analizadas mediante AD.

**Tabla S2.** Efecto de la temperatura en la etapa de elución del ADN

|  | TE | | TE80 | | dNTPs | | dNTPs80 | |
| --- | --- | --- | --- | --- | --- | --- | --- | --- |
| Muestra HN | Ct  *RNasa P* | Ct  IS*481* | Ct  *RNasa P* | Ct  IS*481* | Ct  *RNasa P* | Ct  IS*481* | Ct  *RNasa P* | Ct  IS*481* |
| 1 | 29,12 | 25,93 | 27,75 | 23,15 | 29,33 | 25,12 | 28,91 | 23,50 |
| 2* | 30,29 | 20,80 | 30,66 | 20,99 | 30,84 | 20,64 | 30,19 | 20,73 |
| 3* | 32,49 | 21,61 | 32,14 | 21,95 | 32,78 | 21,96 | 32,89 | 21,93 |

**HN:** hisopado nasofaríngeo. **TE:** elución de ADN en *buffer* TE: a temperatura ambiente. **TE80:** elución de ADN en *buffer* TE con calentamiento a 80°C. **dNTPs:** elución de ADN en solución dNTPs a temperatura ambiente. **dNTPs80:** elución de ADN en solución dNTPs con calentamiento a 80°C. **Ct:** Cycle Threshold.

**Tabla S3.** PCR en tiempo real de ADN de *B. pertussis* purificado por el método rápido y convencional

| **ID muestra** | **Método rápido de purificación**  **basado en disco de celulosa** | | | **Método convencional de purificación**  **basado en columnas de sílice** | | |
| --- | --- | --- | --- | --- | --- | --- |
|  | **Ct**  ***RNasa P*** | **Ct**  **IS*481*** | **Resultado** | **Ct**  ***RNasa P*** | **Ct**  **IS*481*** | **Resultado** |
| 1 | 14,25 | 13,21 | Negativo | 26,60 | 0 | Negativo |
| 2 | 29,59 | 23,58 | Positivo | 25,93 | 19,85 | Positivo |
| 3 | 23,85 | 27,43 | Positivo | 23,49 | 30,96 | Positivo |
| 4 | 23,25 | 34,46 | Positivo | 23,52 | 28,13 | Positivo |
| 5 | 32,79 | 27,03 | Positivo | 36,13 | 17,39 | Positivo |
| 6 | 25,84 | 17,39 | Positivo | 24,30 | 14,38 | Positivo |
| 7 | 30,56 | 20,48 | Positivo | 28,06 | 17,08 | Positivo |
| 8 | 27,59 | 12,94 | Positivo | 29,38 | 14,57 | Positivo |
| 9 | 25,61 | 20,66 | Positivo | 25,88 | 21,53 | Positivo |
| 10 | 31,39 | 29,22 | Positivo | 31,69 | 27,83 | Positivo |
| 11 | 27,53 | 26,54 | Positivo | 30,81 | 27,92 | Positivo |
| 12 | 23,30 | 30,97 | Positivo | 28,90 | 31,48 | Positivo |
| 13 | 24,66 | 21,57 | Positivo | 27,16 | 21,52 | Positivo |
| 14 | 26,40 | 25,09 | Positivo | 30,84 | 25,67 | Positivo |
| 15 | 25,52 | 15,80 | Positivo | 28,39 | 19,33 | Positivo |
| 16 | 35,07 | 31,05 | Positivo | 36,26 | 27,29 | Positivo |
| 17 | 23,37 | 21,60 | Positivo | 27,22 | 23,68 | Positivo |
| 18 | 25,42 | 19,66 | Positivo | 29,14 | 25,48 | Positivo |
| 19 | 26,62 | 27,05 | Positivo | 26,45 | 19,86 | Positivo |
| 20 | 26,13 | 20,28 | Positivo | 26,15 | 18,49 | Positivo |
| 21 | 24,46 | 0 | Negativo | 22,27 | 0 | Negativo |
| 22 | 32,90 | 0 | Negativo | 39,12 | 0 | Negativo |
| 23 | 27,01 | 22,95 | Positivo | 25,22 | 19,08 | Positivo |
| 24 | 26,47 | 0 | Negativo | 23,57 | 41,19 | Negativo |
| 25 | 25,23 | 0 | Negativo | 24,25 | 0 | Negativo |
| 26 | 27,12 | 30,12 | Positivo | 25,93 | 26,31 | Positivo |
| 27 | 22,76 | 18,89 | Positivo | 24,69 | 15,98 | Positivo |
| 28 | 24,75 | 21,81 | Positivo | 25,01 | 15,20 | Positivo |
| 29 | 35,16 | 0 | Negativo | 32,69 | 30,90 | Positivo |
| 30 | 24,75 | 19,41 | Positivo | 22,49 | 14,80 | Positivo |
| 31 | 26,35 | 0 | Negativo | 24,77 | 0 | Negativo |
| 32 | 27,77 | 37,02 | Positivo | 24,79 | 24,23 | Positivo |
| 33 | 25,69 | 18,94 | Positivo | 26,81 | 21,35 | Positivo |
| 34 | 23,67 | 20,80 | Positivo | 28,04 | 0 | Negativo |
| 35 | 30,35 | 0 | Negativo | 30,14 | 0 | Negativo |
| 36 | 25,75 | 0 | Negativo | 26,23 | 0 | Negativo |
| 37 | 26,25 | 23,38 | Positivo | 25,63 | 21,62 | Positivo |
| 38 | 29,59 | 39,44 | Positivo | 26,73 | 26,64 | Positivo |
| 39 | 38,75 | 32,31 | Positivo | 36,54 | 27,17 | Positivo |
| 40 | 27,23 | 14,19 | Positivo | 24,87 | 11,71 | Positivo |
| 41 | 24,39 | 19,46 | Positivo | 30,89 | 19,52 | Positivo |
| 42 | 27,25 | 24,38 | Positivo | 29,45 | 23,19 | Positivo |
| 43 | 30,37 | 25,65 | Positivo | 30,44 | 30,06 | Positivo |
| 44 | 28,90 | 27,68 | Positivo | 27,88 | 24,77 | Positivo |
| 45 | 0 | 0 | Negativo | 0 | 0 | Negativo |
| 46 | 24,91 | 19,66 | Positivo | 23,26 | 16,28 | Positivo |
| 47 | 26,85 | 16,81 | Positivo | 25,71 | 24,80 | Positivo |
| 48 | 30,59 | 0 | Negativo | 28,44 | 0 | Negativo |
| 49 | 26,96 | 18,32 | Positivo | 24,43 | 14,38 | Positivo |
| 50 | 34,02 | 30,09 | Positivo | 26,31 | 17,81 | Positivo |
| 51 | 35,12 | 30,30 | Positivo | 30,26 | 26,87 | Positivo |
| 52 | 27,96 | 20,58 | Positivo | 29,32 | 19,19 | Positivo |
| 53 | 28,96 | 25,77 | Positivo | 0 | 40,18 | Positivo |
| 54 | 38,98 | 0 | Negativo | 25,71 | 38,33 | Positivo |
| 55 | 26,73 | 29,68 | Positivo | 26,33 | 22,49 | Positivo |
| 56 | 30,10 | 33,00 | Positivo | 25,36 | 30,30 | Positivo |
| 57 | 30,12 | 28,00 | Positivo | 27,32 | 20,43 | Positivo |
| 58 | 27,21 | 37,91 | Positivo | 25,26 | 26,06 | Positivo |
| 59 | 25,13 | 12,65 | Positivo | 22,52 | 8,71 | Positivo |
| 60 | 29,60 | 0 | Negativo | 24,45 | 0 | Negativo |
| 61 | 32,70 | 34,03 | Positivo | 30,57 | 29,42 | Positivo |
| 62 | 25,33 | 22,39 | Positivo | 22,55 | 15,22 | Positivo |
| 63 | 33,48 | 0 | Negativo | 22,67 | 24,21 | Positivo |
| 64 | 28,83 | 16,59 | Positivo | 26,51 | 12,11 | Positivo |
| 65 | 31,63 | 27,90 | Positivo | 31,20 | 22,99 | Positivo |
| 66 | 31,53 | 25,56 | Positivo | 32,19 | 23,51 | Positivo |
| 67 | 29,96 | 16,60 | Positivo | 0 | 14,69 | Positivo |
| 68 | 37,07 | 30,55 | Positivo | 41,29 | 28,37 | Positivo |
| 69 | 28,12 | 38,55 | Positivo | 27,35 | 26,34 | Positivo |
| 70 | 0 | 32,50 | Positivo | 0 | 22,79 | Positivo |
| 71 | 32,01 | 0 | Negativo | 25,15 | 25,33 | Positivo |
| 72 | 36,75 | 0 | Negativo | 0 | 0 | Negativo |
| 73 | 25,82 | 24,32 | Positivo | 25,89 | 18,19 | Positivo |
| 74 | 27,48 | 16,11 | Positivo | 25,50 | 11,31 | Positivo |
| 75 | 36,07 | 0 | Negativo | 29,47 | 0 | Negativo |
| 76 | 25,74 | 23,39 | Positivo | 27,55 | 18,18 | Positivo |
| 77 | 39,90 | 0 | Negativo | 30,30 | 29,41 | Positivo |
| 78 | 30,26 | 0 | Negativo | 29,20 | 0 | Negativo |
| 79 | 31,01 | 0 | Negativo | 29,94 | 29,04 | Positivo |
| 80 | 29,95 | 27,04 | Positivo | 27,53 | 17,78 | Positivo |
| 81 | 29,15 | 30,36 | Positivo | 29,09 | 22,39 | Positivo |
| 82 | 0 | 21,38 | Positivo | 23,69 | 15,80 | Positivo |
| 83 | 27,83 | 26,05 | Positivo | 30,63 | 16,73 | Positivo |
| 84 | 29,78 | 34,79 | Positivo | 26,52 | 24,77 | Positivo |
| 85 | 0 | 0 | Negativo | 32,40 | 0 | Negativo |
| 86 | 27,50 | 15,00 | Positivo | 0 | 9,52 | Positivo |
| 87 | 29,42 | 19,89 | Positivo | 19,41 | 10,59 | Positivo |
| 88 | 26,90 | 23,84 | Positivo | 23,61 | 17,00 | Positivo |
| 89 | 28,20 | 28,46 | Positivo | 30,74 | 24,86 | Positivo |
| 90 | 30,53 | 22,08 | Positivo | 0 | 14,31 | Positivo |
| 91 | 27,95 | 23,69 | Positivo | 24,55 | 17,55 | Positivo |
| 92 | 32,15 | 39,50 | Positivo | 28,31 | 27,27 | Positivo |
| 93 | 30,82 | 0 | Negativo | 25,41 | 21,73 | Positivo |
| 94 | 28,85 | 0 | Negativo | 25,25 | 0 | Negativo |
| 95 | 33,60 | 0 | Negativo | 27,47 | 0 | Negativo |
| 96 | 31,60 | 0 | Negativo | 27,08 | 31,23 | Positivo |
| 97 | 26,18 | 20,83 | Positivo | 0 | 15,56 | Positivo |
| 98 | 26,94 | 21,32 | Positivo | 26,40 | 16,20 | Positivo |
| 99 | 27,80 | 34,27 | Positivo | 30,20 | 34,79 | Positivo |
| 100 | 32,59 | 0 | Negativo | 22,77 | 22,29 | Positivo |
| 101 | 24,82 | 0 | Negativo | 19,33 | 0 | Negativo |
| 102 | 32,08 | 0 | Negativo | 25,59 | 0 | Negativo |
| 103 | 23,74 | 0 | Negativo | 18,54 | 0 | Negativo |
| 104 | 33,36 | 0 | Negativo | 27,48 | 0 | Negativo |
| 105 | 27,96 | 0 | Negativo | 20,92 | 0 | Negativo |
| 106 | 26,49 | 0 | Negativo | 21,62 | 0 | Negativo |
| 107 | 27,24 | 0 | Negativo | 22,46 | 0 | Negativo |
| 108 | 23,71 | 0 | Negativo | 19,48 | 0 | Negativo |
| 109 | 24,84 | 0 | Negativo | 18,77 | 0 | Negativo |
| 110 | 34,62 | 0 | Negativo | 27,00 | 0 | Negativo |
| 111 | 27,82 | 0 | Negativo | 24,31 | 0 | Negativo |
| 112 | 25,44 | 0 | Negativo | 26,16 | 0 | Negativo |
| 113 | 25,54 | 0 | Negativo | 24,92 | 0 | Negativo |
| 114 | 24,71 | 0 | Negativo | 21,02 | 0 | Negativo |
| 115 | 23,10 | 0 | Negativo | 20,73 | 0 | Negativo |
| 116 | 23,10 | 0 | Negativo | 21,86 | 0 | Negativo |
| 117 | 23,38 | 0 | Negativo | 22,07 | 0 | Negativo |
| 118 | 27,09 | 0 | Negativo | 24,99 | 0 | Negativo |
| 119 | 23,60 | 0 | Negativo | 23,36 | 0 | Negativo |
| 120 | 25,28 | 0 | Negativo | 22,51 | 0 | Negativo |
| 121 | 24,92 | 0 | Negativo | 21,45 | 0 | Negativo |
| 122 | 27,67 | 0 | Negativo | 24,04 | 0 | Negativo |
| 123 | 25,57 | 0 | Negativo | 21,12 | 0 | Negativo |
| 124 | 24,37 | 0 | Negativo | 20,36 | 0 | Negativo |
| 125 | 23,99 | 0 | Negativo | 19,41 | 0 | Negativo |
| 126 | 26,38 | 0 | Negativo | 22,23 | 0 | Negativo |
| 127 | 24,17 | 0 | Negativo | 19,49 | 0 | Negativo |
| 128 | 25,12 | 0 | Negativo | 20,41 | 0 | Negativo |
| 129 | 26,20 | 0 | Negativo | 21,71 | 0 | Negativo |
| 130 | 26,58 | 0 | Negativo | 22,12 | 0 | Negativo |
| 131 | 25,65 | 0 | Negativo | 24,56 | 0 | Negativo |
| 132 | 24,35 | 0 | Negativo | 21,91 | 0 | Negativo |
| 133 | 26,09 | 0 | Negativo | 22,83 | 0 | Negativo |
| 134 | 29,18 | 0 | Negativo | 25,80 | 0 | Negativo |
| 135 | 24,24 | 0 | Negativo | 19,84 | 0 | Negativo |
| 136 | 27,15 | 0 | Negativo | 22,95 | 0 | Negativo |
| 137 | 27,61 | 0 | Negativo | 24,33 | 0 | Negativo |
| 138 | 26,29 | 0 | Negativo | 23,17 | 0 | Negativo |
| 139 | 29,76 | 0 | Negativo | 25,74 | 0 | Negativo |
| 140 | 25,72 | 0 | Negativo | 22,86 | 0 | Negativo |
| 141 | 27,45 | 0 | Negativo | 21,95 | 0 | Negativo |
| 142 | 26,44 | 0 | Negativo | 23,63 | 0 | Negativo |
| 143 | 26,58 | 0 | Negativo | 24,04 | 0 | Negativo |
| 144 | 23,45 | 0 | Negativo | 18,65 | 0 | Negativo |
| 145 | 22,31 | 0 | Negativo | 21,28 | 0 | Negativo |
| 146 | 25,13 | 0 | Negativo | 22,22 | 0 | Negativo |
| 147 | 26,72 | 0 | Negativo | 23,02 | 0 | Negativo |
| 148 | 24,30 | 0 | Negativo | 19,97 | 0 | Negativo |
| 149 | 27,96 | 0 | Negativo | 24,59 | 0 | Negativo |
| 150 | 29,75 | 0 | Negativo | 23,89 | 0 | Negativo |

**Ct:** *Cycle Threshold*

**Figuras**


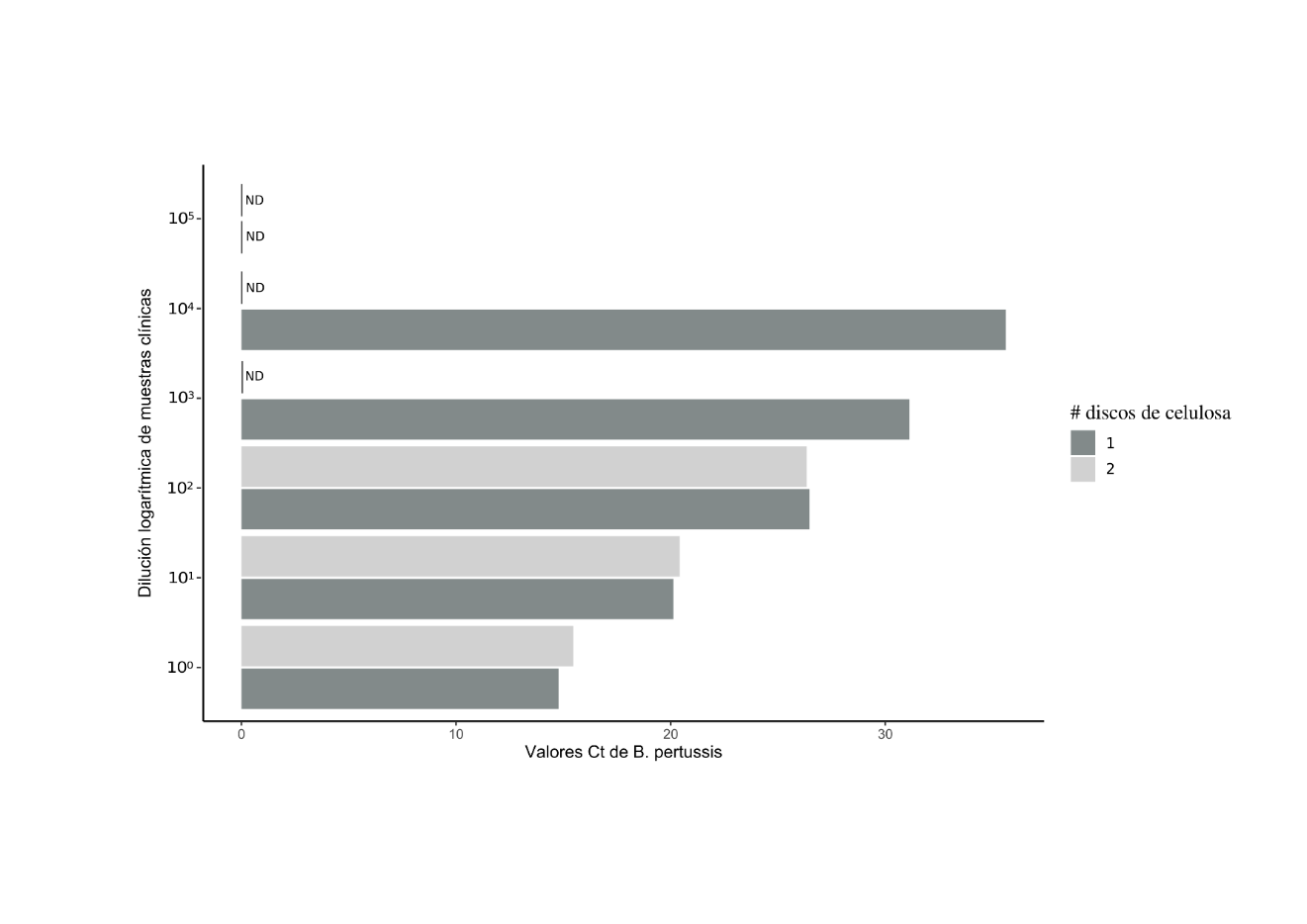


**Ct:** *Cycle Threshold*. **ND:** no se detectó ADN de *B. pertussis* por qPCR.

**Figura S1.** Valores de Ct de PCR en tiempo real de ADN de *B. pertussis* purificado por el método rápido utilizando 1 y 2 discos de celulosa. El ADN fue obtenido de diluciones de hisopados nasofaríngeos de individuos que resultaron positivos para ADN de *B. pertussis* por qPCR.
