# Supplementary material for: Direct amplification of Bordetella pertussis DNA purified from nasopharyngeal swabs by a low-cost, fast (60-second), and equipment-free method
Source: Rev Peru Med Exp Salud Publica. 2022 Sep 30;39(3):312–20. doi: 10.17843/rpmesp.2022.393.10865 (PMC11397763; doi:10.17843/rpmesp.2022.393.10865)
Supplement: Translation

# Translation: Amplificación directa de ADN de *Bordetella pertussis *purificado de hisopados nasofaríngeos por un método de bajo costo, rápido (60-segundos) y libre de equipos

**Authors:** Eduardo Juscamayta-López, Faviola Valdivia, María Pía Soto, Helen Horna, Brenda Nureña

**Affiliations:** 1 Laboratorio de Infecciones Respiratorias Agudas, Centro Nacional de Salud Pública, Instituto Nacional de Salud, Lima, Perú.

**Keywords:** Pruebas en el punto de atención, Aislamiento & purificación, ADN, *Bordetella pertussis*, Celulosa, Técnicas de diagnóstico molecular, Reacción en cadena en tiempo real de la polimerasa, Amplificación Basada en la Secuencia del Ácido Nucleico, Tos ferina

## Abstract

**Objetivo.:**

Desarrollar y evaluar un método de bajo costo basado en celulosa para la purificación rápida y amplificación directa de ADN de *Bordetella pertussis* de hisopados nasofaríngeos.

**Materiales y métodos.:**

Se prepararon discos de celulosa y se evaluaron diferentes parámetros (*buffers* de lisis/lavado, número de discos y elución de ADN). El método se acopló a una amplificación directa por PCR en tiempo real (qPCR) y se estimó el rendimiento utilizando hisopados nasofaríngeos que fueron positivos (n=100) y negativos (n=50) para ADN *B. pertussis* por qPCR, comparado con el método basado en columnas de sílice. Se calculó el grado de concordancia, sensibilidad, especificidad, valor predictivo positivo (VPP) y valor predictivo negativo (VPN). Se evaluó la factibilidad del método rápido para ser acoplado a un ensayo colorimétrico de amplificación isotérmica mediada por lazo (LAMP).

**Resultados.:**

El método rápido con un disco de celulosa y *buffer* de lisis y lavado conteniendo PVP-40 y *Tween *20, respectivamente, mostró una mayor capacidad para purificar ADN amplificable de *B. pertussis.* El método tuvo una sensibilidad de 89,0% (IC95%, 80,2%-94,9%) y una especificidad de 98,5% (IC95%, 92,1%-100,0%), con un buen grado de concordancia (Kappa=0,867; IC95% 0,788 - 0,946), respecto al método referencial. Los VPP y VPN fueron 98,6% (IC95%, 92,7,2%-100,0%) y 88,2% (IC95%, 78,7%-94,4%), respectivamente. Se evidenció una amplificación exitosa por LAMP, y se obtuvieron resultados comparables con el método por columnas de sílice.

**Conclusión.:**

El método desarrollado es simple, de bajo costo y libre de equipos para la obtención rápida (60 segundos) de ADN en el punto de atención, y puede ser implementado en diversas técnicas moleculares orientados al diagnóstico oportuno y al estudio epidemiológico de tos ferina.

## INTRODUCCIÓN

La tos ferina o pertussis es una infección respiratoria altamente trasmisible causada por la bacteria *Bordetella pertussis; *es una enfermedad de gran importancia para la salud pública a nivel global debido a la severidad que puede presentar; afecta especialmente a niños menores de cinco años, mayormente a los neonatos ^1^.

En el Perú, la tos ferina es considerada una enfermedad endémica con picos de infección cada 3 a 5 años ^2^, por lo que un método de diagnóstico rápido y altamente sensible es la clave para controlar la enfermedad y limitar su transmisión. El diagnóstico molecular de pertussis se basa en la reacción en cadena de la polimerasa en tiempo real (qPCR) utilizando ADN obtenido de hisopados nasofaríngeos ^3^. Sin embargo, la purificación de ácidos nucleicos de muestras clínicas es una compleja tarea que demanda tiempo, personal especializado y recursos, que convierte a este procedimiento en un cuello de botella que retrasa el diagnóstico de la enfermedad ^4^.

La constante búsqueda por nuevos métodos de purificación de ácidos nucleicos propone actualmente un abanico de posibilidades, donde los kits de extracción de fase sólida son los más utilizados para posteriores aplicaciones moleculares ^5^. La mayor parte de estos kits comerciales se basan en columnas con membranas de sílice y se fundamenta en la interacción de cargas de los ácidos nucleicos y la matriz, bajo estrictas condiciones alcalinas, generando así una unión selectiva de los ácidos nucleicos ^6^ ^) ^y posterior obtención del material genético; no obstante, el proceso demanda tiempo debido a sus numerosas etapas, así como el uso de equipos y personal capacitado ^7^. La tecnología de perlas magnéticas se ha convertido en un proceso de purificación muy empleado en las últimas décadas, en el cual, por hibridación complementaria, los ácidos nucleicos se unen a partículas magnéticas, que posteriormente son inmovilizadas, permitiendo la remoción de contaminantes ^8^. Aunque este método es relativamente rápido (entre 15 a 30 min) y no necesita centrifugación, su aplicación fuera del laboratorio es un desafío ^9^. Además, los residuos de perlas magnéticas, o su uso directo en la PCR, puede inhibir el proceso de amplificación conduciendo a una menor sensibilidad o falsos negativos ^10^.

Otros métodos de extracción de ácidos nucleicos basados en membranas, como las tarjetas *Flinders Technology Associates* (FTA) y los filtros de sílice Fusión 5, han mostrado resultados prometedores en diferentes muestras biológicas ^11^ ^-^ ^13^. La ventaja de estos métodos radica en la eliminación de la etapa de elución del material genético y la directa amplificación de los ácidos nucleicos de las membranas ^13^. No obstante, demandan múltiples pasos en su proceso y el uso de equipos, por lo que se descartan como métodos simples y rápidos ^14^. 

Otras técnicas basadas en celulosa han demostrado ser útiles en la obtención de ácidos nucleicos de diferentes organismos, incluyendo tejidos vegetales ^15^ ^) ^ y animales ^16^; así como de líneas celulares ^17^. La presente investigación tuvo como objetivo desarrollar y evaluar un método de bajo costo basado en un disco de celulosa para la purificación rápida (60 s) y amplificación directa de ADN de *Bordetella pertussis* de hisopados nasofaríngeos.

**MENSAJE CLAVE**

Motivación para realizar el estudio: la tos ferina continúa siendo una enfermedad endémica con mayor severidad en infantes, por ello son necesarias herramientas moleculares que reduzcan el tiempo y costo de obtención de ADN de* B. pertussis *para su detección rápida y contención de la transmisibilidad.

Principales hallazgos: el método rápido de extracción de ADN demostró un buen nivel de concordancia frente a la purificación por columnas de sílice, obteniendo ADN listo para amplificación directa en 60 s.

Implicancias: el método desarrollado es simple, de bajo costo y libre de equipos para la obtención de ADN, que permitirá el diagnóstico oportuno y la vigilancia molecular de tos ferina en lugares con recursos limitados.

## MATERIALES Y MÉTODOS

### Diseño de estudio y colección de las muestras

La presente investigación es un estudio retrospectivo para desarrollar y evaluar un método basado en un disco de celulosa para la purificación rápida y amplificación directa de ADN de *B. pertussis* utilizando hisopados nasofaríngeos obtenidos de casos de tos ferina confirmados por PCR en tiempo real por el Laboratorio de Referencia Nacional de Infecciones Respiratorias Agudas del Instituto Nacional de Salud (LRNIRA-INS), durante el periodo de 2018 a 2019.

### Preparación de discos de celulosa

Se prepararon discos de celulosa de 6 mm de diámetro de papel Whatman 2 (GE Healthcare) utilizando un perforador, los cuales fueron posteriormente esterilizados por calor seco y almacenados a temperatura ambiente hasta su uso. 

### Evaluación de *buffer* de lisis y de lavado

Con el fin de determinar los mejores *buffers*, se evaluaron dos soluciones de lisis y de lavado (Tabla 1) utilizando muestras clínicas positivas para ADN de *B. pertussis* por qPCR y con valores de Ct (*Cycle Threshold*) de 24,71 y 24,47, respectivamente. Para cada espécimen clínico se realizaron diluciones seriales por duplicado con *buffer* fosfato salino (PBS, por sus siglas en inglés) hasta 10^-5^. En resumen, la metodología consistió en mezclar 100 μL de muestra en 200 μL de *buffer* de lisis, agitar suavemente el lisado y colocar el disco de celulosa por 15 s en la solución, luego trasladar el disco a 200 μL de *buffer* de lavado por 1 min y, finalmente, transferir el disco a la mezcla de amplificación.

**Tabla 1:** Composición de los buffers de lisis y lavado.

*Buffer*	Composición	Referencia
BLS#1	20 mM Tris [pH8], 25 mM NaCl, 2,5 mM EDTA, 0,05% SDS, 2% PVP-40	Mason y Botella, 2020
BLS#2	20 mM Tris [pH8], 25 mM NaCl, 2,5 mM EDTA, 0,05% SDS	Mason y Botella, 2019
BLV#1	10 mM Tris [pH8], 0,1% Tween 20	Zou *et al.*, 2017
BLV#2	10 mM Tris [pH8]	Mason y Botella, 2019

BLS: buffer de lisis. BLV: buffer de lavado. NaCl: cloruro de sodio. EDTA: ácido etilendiaminotetraacético. SDS: dodecilsulfato sódico. PVP-40: polivinilpirrolidona.

A fin de evaluar la pérdida de material genético contenido en el disco de celulosa por un tiempo prolongado de incubación en el *buffer* de lavado, se agregó 100 μL de muestra a 200 μL de *buffer* de lisis 1 (BLS#1), se agitó la solución en v**ó**rtex durante unos segundos y se colocó el disco de celulosa por 1 min, realizando una ligera agitación manual; luego se trasladó el disco a 200 μL de *buffer* de lavado 1 (BLV#1) donde permaneció por 18 h a 4 °C.

### Evaluación del número de discos de celulosa

Se decidió evaluar si el uso de más de un disco de celulosa podría mejorar la obtención de ADN y la sensibilidad de detección del método rápido de purificación. Para tal fin se creó un panel de diluciones a partir de una muestra de hisopado nasofaríngeo positiva para ADN de *B. pertussis* con Ct=21,71. La muestra fue diluida en PBS por duplicado hasta 10^-5^. La purificación del material genético de cada dilución fue obtenida según el procedimiento ya mencionado, incluyendo uno o dos discos de celulosa por muestra, según corresponda. El ADN obtenido por el método rápido de purificación, en ambos casos, fue analizado por PCR en tiempo real basado en la secuencia de inserción 481 (IS*481*).

### Elución del ADN capturado en el disco de celulosa

Se evaluó la etapa de elución del ADN utilizando: (1) Tris-ácido etilendiaminotetraacético (TE: 10 mM Tris, pH 8,5, 0,1 mM EDTA) y (2) solución de dNTPs (400µM). El proceso se realizó por duplicado, mezclando 100 μL de muestra en 200 μL de BLS#1, se agitó la solución en vórtex durante unos segundos y se colocó el disco de celulosa por 1 min realizando una ligera agitación manual, luego se trasladó el disco a 200 μL de BLV#1 por 1 min. Finalizada la etapa de lavado, se transfirió un disco a la mezcla de amplificación y el otro, dividido en dos, a 50 μL de cada solución de elución y se incubó por 5 min a temperatura ambiente. En un posterior ensayo se evaluó el efecto de un tratamiento con calor, para lo cual se probaron dos condiciones: incubación del disco en solución de elución a temperatura ambiente y con calentamiento a 80 °C, ambas por 5 min.

### Purificación de ácidos nucleicos utilizando kit comercial basado en columnas de sílice

Para la purificación de ADN de las muestras de hisopados nasofaríngeos, se utilizó el kit de purificación *PureLink Genomic DNA Mini Kit* (Invitrogen), siguiendo las especificaciones del fabricante. En resumen, se utilizó 200 μL de muestra, siguiendo las etapas de digestión con proteinasa K a 55 °C por 1 h, lisis y lavado con sus respectivos *buffers*. El ADN fue obtenido en 100 μL de tampón de elución y almacenado a 4 °C para su posterior análisis por qPCR.

### Purificación rápida de ácidos nucleicos basado en disco de celulosa y amplificación directa

El método de extracción optimizado consistió en mezclar 100 μL de muestra de hisopado nasofaríngeo con 200 μL de BLS#1. La mezcla se agitó en vórtex por 15 s y se agregó el disco de celulosa. Luego, con la ayuda de una punta estéril de 1000 μL se trasladó el disco a 200 μL de BLV#1, se agitó suavemente por unos segundos y se dejó incubar a temperatura ambiente por 1 min. Finalmente, el disco se colocó en la mezcla de amplificación, previamente preparada (Figura 1).

**Figura 1:** Flujo del método rápido de purificación acoplada a la amplificación directa de ADN de* B. pertussis.* ADN total obtenido por un método simple y rápido (60 s) basado en disco de celulosa y disponible para análisis moleculares qPCR o LAMP.

### Amplificación directa mediante PCR en tiempo real y LAMP

Para comparar el rendimiento del nuevo método rápido con el método convencional de extracción de ADN, se utilizaron 150 muestras clínicas que fueron positivas (n=100) y negativas (n=50) para ADN *B. pertussis* por qPCR. El ADN genómico de estas muestras fue extraído mediante un kit comercial y el método basado en el disco de celulosa de forma paralela y evaluado por PCR en tiempo real. Los ensayos de PCR en tiempo real se realizaron de acuerdo con la metodología descrita por Tatti *et al*. (2011), que consiste en un ensayo multiplex para detección de IS*481*, pIS*1001* y hIS*1001* y el gen *RNasa* P como control interno. La amplificación se realizó en el termociclador Rotor-Gene Q (Qiagen), utilizando los siguientes parámetros de ciclo: 50 °C durante 2 min, activación de la enzima a 95 °C durante 10 min y 45 ciclos de amplificación a 95 °C durante 15 s y 60 °C durante 1 min.

Los ensayos LAMP se llevaron a cabo con el reactivo de amplificación de ARN/ADN Loopamp D (Eiken Chemical Co., Ltd., Tokio, Japón) y se utilizaron cebadores dirigidos a la región MaoC (Juscamayta *et al*., data no publicada). La reacción LAMP consistió de un volumen total de 25 μL con 40 pmol de cebadores internos (FIP y BIP), 5 pmol de cebadores externos (F3 y B3), 20 pmol de cebadores formadores de bucle (LF y LB) y el disco de celulosa. Los tubos se incubaron a 65 °C durante 60 min y a 80 °C por 5 min para inactivar la reacción. Los resultados fueron analizados visualmente, considerándose una muestra positiva por el viraje del color marrón a verde en la solución de reacción.

### Análisis estadístico

Se utilizaron los programas R v4.0.5 y Stata/MP v15.0 para ejecutar el análisis estadístico. Los valores de Ct fueron comparados mediante análisis de varianza de una vía (ANOVA), previa evaluación de la normalidad utilizando la prueba de Shapiro-Wilk. Los resultados positivos y negativos de PCR en tiempo real de ADN obtenidos por el método rápido de purificación y basado en columnas de sílice fueron analizados en una tabla de contingencia 2x2. El análisis se realizó utilizando coeficientes de concordancia kappa ^18^. Se calcularon los intervalos de confianza (IC) del 95 %, y p<0,05 bilateral se consideró significativo para todos los análisis estadísticos.

### Aspectos éticos

El presente estudio se llevó a cabo bajo el protocolo de investigación aprobado por el Comité Institucional de Ética en Investigación del INS (código: OI-032-18).

## RESULTADOS

### Evaluación de *buffer* de lisis y lavado, y número de discos de celulosa

La evaluación de los *buffers* de lisis y lavado resultó en valores menores de Ct para la combinación de BLS#1 basado en PVP-40 y BLV#1 conteniendo *Tween *20. El método rápido de extracción de ADN utilizando BLS#1 y BLS#2 logró purificar ADN de *B. pertussis* de una muestra clínica diluida hasta 10^-4^ y 10^-3^, respectivamente; mientras que con el *buffer* de lavado se logró purificar ADN de *B. pertussis* hasta 10^-5^ y 10^-4^ utilizando BLV#1 y BLV#2, respectivamente. A pesar de que se encontraron ligeras diferencias entre los valores de Ct obtenidos con los *buffers* de lisis para las diluciones 10^-1^ hasta 10^-3^, estas diferencias no fueron estadísticamente significativas (p>0,05) (Figura 2). Por el contrario, la diferencia entre los Cts alcanzados con los *buffers* de lavado fue estadísticamente significativa para las diluciones entre 10^-2^ y 10^-4^ (Figura 3).

**Figura 2:** Valores de Ct de PCR en tiempo real de ADN de *B. pertussis *purificado con diferentes soluciones de lisis. El ADN fue obtenido de diluciones de hisopados nasofaríngeos de individuos que resultaron positivos para ADN de *B. pertussis* por qPCR.

**Figura 3:** Valores de Ct de PCR en tiempo real de ADN de *B. pertussis* purificado con diferentes soluciones de lavado. El ADN fue obtenido de diluciones de hisopados nasofaríngeos de individuos que resultaron positivos para ADN de *B. pertussis* por qPCR.

La exposición del disco de celulosa por un tiempo prolongado (18 h) en *buffer* de lavado, no afectó la detección de ADN de *B. pertussis* mediante PCR en tiempo real, no observándose diferencias significativas entre los valores de Cts para IS*481* y* RNasa P *obtenidos después de la incubación del disco por 1 min (Ct_*IS481*_=16,42-20,99 y Ct_*RnasaP*_=25,31-30,84) y la exposición por 18 h (Ct_*IS481*_=17,21-21,96 y Ct_*RnasaP*_=27,11-32,89). Asimismo, al trabajar con dos discos de celulosa por muestra clínica, el método rápido fue capaz de detectar ADN de *B. pertussis* hasta una dilución de 10^-2 ^(Ct=15,46-26,34), mientras que utilizando un disco se logró detectar ADN de *B. pertussis* hasta la dilución 10^-4^ (Ct= 4,77-35,62) (Figura S1).

### Elución del ADN capturado en el disco de celulosa

Con el fin de verificar la presencia de ADN en el disco de celulosa, se llevaron a cabo ensayos de purificación rápida utilizando soluciones de elución incluyendo *buffer* TE y solución de dNTPs, que fueron comparados con el método de purificación rápida y amplificación directa sin elución. Ambas soluciones fueron capaces de eluir ADN de *B. pertussis* del disco de celulosa, resultando en valores similares de Cts para IS*481*, con una mediana de 23,94 y 26,56 para TE y dNTPs, respectivamente (Tabla S1). El método de purificación rápida acoplada a la amplificación directa sin elución resultó en valores más bajos de Cts que los obtenidos con TE y dNTPs, tanto para el gen de control interno como la diana específica de *B. pertussis*. (Tabla S1). Al adicionar un tratamiento de temperatura a la etapa de elución, no se observaron diferencias significativas entre los valores de Cts para IS*481* y* RNasa P* de cada *buffer* de elución evaluado (Tabla S2).

### Evaluación del método rápido de purificación de ADN basado en celulosa

La sensibilidad, especificidad, valor predictivo positivo y valor predictivo negativo de la metodología rápida de purificación y amplificación directa de ADN de *B. pertussis* resultó en 89,0% (IC95%, 80,2%-94,9%), 98,5% (IC95%, 92,1%-100,0%), 98,6% (IC95%, 92,7%-100,0%) y 88,2% (IC95%, 78,7%-94,4%), respectivamente, comparado con el método de extracción basado en columnas de sílice y amplificación por qPCR (metodología de referencia) (Figura 4, Tabla S3). El método de extracción convencional logró detectar ADN de *B. pertussis* en el 82% de las muestras clínicas positivas (82/100), mientras que con el método rápido se obtuvo ADN de *B. pertussis* en el 74% de los especímenes positivos (74/100). De las 68 muestras negativas para ADN de *B. pertussis* obtenidas por el método convencional, 67 también fueron negativas por el método rápido, generando 1 falso positivo. Asimismo, de las 82 muestras positivas para ADN de *B. pertussis *purificadas con el método convencional, 73 fueron positivas por el método rápido basado en celulosa, generando nueve falsos negativos. El grado de concordancia entre los resultados de ambos métodos resultó en un coeficiente de kappa de 0,867 (IC95% 0,788 - 0,946).

**Figura 4:** Curvas de amplificación para IS481 de ADN de *B. pertussis* de muestras de hisopado nasofaríngeo purificado por el método rápido (A) y convencional (B).

### Amplificación directa utilizando la metodología isotérmica LAMP

Con el fin de evaluar la factibilidad del método rápido de purificación acoplado a la amplificación directa con un método molecular rápido y de bajo costo, se realizó un ensayo de amplificación isotérmica mediada por lazo (LAMP), utilizando una muestra clínica positiva para ADN de *B. pertussis* por qPCR. El ADN fue purificado nuevamente del espécimen clínico por el método rápido y convencional, seguido de amplificación mediante LAMP y PCR en tiempo real, respectivamente. El ensayo LAMP logró detectar de manera específica ADN de *B. pertussis* obtenido por el método rápido que se visualizó a través del cambio del color, de marrón a verde (Figura 5A). Similar resultado se observó en el ensayo de PCR en tiempo real, que fue capaz de detectar ADN de *Bordetella pertussis *(Ct=12,79) purificado por el método convencional en la misma muestra evaluada (Figura 5B). No se evidenció amplificación en los controles negativos en ninguno de los ensayos (Figura 5).

**Figura 5:** Amplificación directa LAMP de ADN de *B. pertussis* purificado por el método rápido basado en celulosa. Las reacciones fueron consideradas positivas para producto LAMP si estas cambiaron de color de marrón a verde (A). El ADN fue obtenido de una muestra de hisopado nasofaríngeo por el método rápido y convencional y analizadas mediante LAMP (A) y PCR en tiempo real (B), respectivamente.

## DISCUSIÓN

Los métodos moleculares rápidos y simples son necesarios para potenciar el diagnóstico y controlar la propagación de enfermedades infecciosas altamente contagiosas como la tos ferina. Sin embargo, la dificultad principal de la detección molecular es que requiere la purificación de ácidos nucleicos lo cual demanda tiempo y el uso de reactivos y equipos costosos ^19^ ^) ^que, además, pueden ser escasos durante periodos de brotes, como lo ha demostrado la pandemia de COVID-19. En este estudio demostramos la amplificación directa de ADN de *B. pertussis* purificado de hisopados nasofaríngeos por un método de bajo costo, rápido (60 s) y sin el empleo de equipo sofisticado.

Existen trabajos que han evaluado diferentes componentes de los *buffers* de extracción de ácidos nucleicos de bacterias, parásitos, hongos y virus ^15^ ^-^ ^17^ ^,^ ^20^. En el presente estudio, la elección de los *buffers* de lisis y lavado se centró en la capacidad de purificar los ácidos nucleicos de los especímenes de hisopados nasofaríngeos diluidos. El *buffer* de lisis conteniendo PVP-40 logró purificar ADN de *B. pertussis* a una mayor dilución que fue detectado por qPCR (Figura 2). El PVP-40 es un agente químico no tóxico que ayuda a minimizar el efecto causado por compuestos interferentes en la amplificación del ADN ^21^. Además, se ha demostrado su efectividad en diferentes tipos de muestras ^17^. Por otro lado, el *buffer* de lavado a base de *Tween* 20 fue el que mostró una mayor sensibilidad analítica (Figura 3), contrariamente al estudio de Mason y Botella ^15^, donde se reportó una interferencia negativa de este compuesto en las reacciones de amplificación. 

El método rápido utilizando solo un disco de celulosa fue capaz de purificar ADN amplificable de *B. pertussis* de una muestra clínica dos veces más diluida, que utilizando dos discos de celulosa (Figura S1). Resultados similares fueron reportados por Zou *et al*., quienes lograron detectar ADN de *Actinobacillus pleuropneumoniae* purificado por disco de celulosa y demostraron que la cantidad y rendimiento del ADN purificado disminuye a mayor número de discos, probablemente por darse una mayor adsorción de los componentes de la PCR en las membranas de celulosa ^17^.

La elución del material genético en soluciones independientes no se vio reflejada en un mejor rendimiento durante la amplificación, por el contrario, la liberación directa del disco de celulosa en la mezcla de amplificación resultó en menores valores de Ct (Tabla S1), lo que sugería una mayor cantidad de ácidos nucleicos recuperados, donde el factor influyente probablemente fueron los dNTPs, que cumplen una función importante en la desorción del ADN de diferentes matrices, como lo demostró Tanaka *et al.*, al comparar el rendimiento de recuperación del ADN extraído con nanopartículas magnéticas modificadas con aminosilano, concluyendo que el uso de dNTPs resulta en una elución muy eficiente, al ser los dNTPs componentes integrales de la amplificación por PCR ^22^. Estudios previos han demostrado que un tratamiento térmico conlleva a una mayor eficiencia de elución del ADN, resultando en una mejor detección por qPCR ^23^, sin embargo, los resultados obtenidos se contraponen a estos, al no observarse diferencias significativas con el tratamiento de temperatura (Tabla S2), por lo que se decidió omitir la etapa de elución, obteniendo una metodología simple y rápida compuesta de solo tres etapas (Figura 1).

El ADN purificado por disco de celulosa mostró un grado de concordancia casi perfecto (kappa=0,867; IC95% 0,788 - 0,946) con los resultados obtenidos por el método basado en columnas de sílice (Figura 4), según la clasificación de Landis y Koch ^24^. De las cien muestras clínicas positivas evaluadas, 18 resultaron negativas para ADN de *B. pertussis* utilizando la metodología basada en columnas de sílice, mientras que con el método rápido se obtuvieron 26 muestras negativas, de las cuales 17/26 coincidieron con los resultados del método convencional. La relativa alta cantidad de falsos negativos podría deberse a que la integridad del material genético se haya visto afectada por los ciclos de congelamiento-descongelamiento de las muestras clínicas almacenadas, resultando en un sesgo en la amplificación con un aumento de falsos negativos en la detección molecular del patógeno ^25^.

La metodología rápida resultó en una sensibilidad y especificidad de 89,0% (IC95%, 80,2%-94,9%) y 98,5% (IC95%, 92,1%-100,0%), respectivamente (Tabla S3). Un estudio de amplificación por recombinasa utilizando ADN de *B. pertussis* purificado por *boiling* mostró una sensibilidad del 85% ^26^, sin embargo, al ser esta una técnica basada únicamente en calor, algunos componentes inhibidores de la PCR no llegan a ser eliminados de las muestras afectando negativamente la reacción de amplificación ^27^. Adicionalmente, la purificación por *boiling* requiere cerca de 15 min, mientras que nuestro método rápido por celulosa, 60 s. Xu *et al.* proponen un método de purificación basado en nanopartículas magnéticas (NPM) modificadas, el cual mostró resultados de amplificación concordantes con aquellos obtenidos por un kit comercial en muestras de sangre humana ^28^. Aunque la metodología NPM ha mostrado un buen rendimiento en la obtención de ADN en un tiempo promedio de 10 min, requiere del uso de equipos y la preparación relativamente compleja de nanopartículas. Dispositivos libres de centrifugación han sido desarrollados para la extracción rápida de ADN en 2 min con rendimiento comparable al purificado por un kit comercial ^29^; sin embargo, no están acoplados a una amplificación directa por PCR o LAMP y se limitan al análisis de una sola muestra. Hasta la fecha, no existen reportes de métodos rápidos de purificación y amplificación directa de ADN de *B. pertussis* de hisopados nasofaríngeos; por lo tanto, se propone en el presente estudio una nueva alternativa simple y rápida de purificación de ácidos nucleicos en este tipo de muestras clínicas.

En este estudio, además, el método de purificación rápido fue acoplado a una amplificación directa por amplificación isotérmica mediada por lazo (LAMP); esta es una técnica molecular sensible, rápida y de bajo costo que se ha utilizado ampliamente en la detección de patógenos respiratorios, incluidos influenza ^30^, *B. pertussis* ^31^y SARS-CoV-2 ^32^. Nuestro ensayo evidenció una amplificación exitosa por LAMP, obteniendo resultados comparables con el método por columnas de sílice. Aula *et al.* obtuvieron resultados similares, quienes utilizando tiras de celulosa lograron una amplificación LAMP exitosa hasta una dilución de 2^-13^ de una muestra con huevos de Schistosoma, demostrando la alta sensibilidad del método de purificación rápido acoplado a una amplificación LAMP ^20^. Asimismo, Kellner *et al*. alcanzaron resultados positivos para la detección de SARS-CoV-2 por amplificación LAMP, donde la sensibilidad de la purificación por tira de celulosa resultó idéntica al método por perlas magnéticas ^33^.

Nuestro método de purificación de ácidos nucleicos basado en disco de celulosa presenta las siguientes ventajas: 1) es una metodología rápida que permite la purificación de ADN en 60 s a partir de hisopados nasofaríngeos; 2) es de bajo costo y no requiere equipos complejos; 3) es un método simple (tres pasos) que genera material genético en muy corto tiempo para ser utilizado en diversas técnicas moleculares orientadas al diagnóstico y al estudio epidemiológico de tos ferina (Figura 1); y 4) tiene el potencial para purificar no solo ADN, sino también ARN, y ser escalado para la detección molecular de cualquier patógeno, incluyendo SARS-CoV-2. Estas características convierten al método en una alternativa muy útil para la purificación rápida y detección molecular de ADN de B. pertussis en entornos con mínimas condiciones de equipamiento, reduciendo la necesidad de transporte y almacenamiento de muestras biológicas.

La principal limitación del presente estudio es el uso retrospectivo de muestras clínicas, esto podría afectar la integridad del material genético y como consecuencia repercutir en el rendimiento del método rápido de purificación de ADN. A diferencia del método de purificación basado en columna de sílice, el método basado en celulosa no concentra los ácidos nucleicos en la muestra. Sin embargo, la ventaja del disco de celulosa es que absorbe el material genético de pequeños volúmenes de muestra y permite una purificación rápida^ (^ ^15^.

En conclusión, este estudio demuestra que el método basado en disco de celulosa permite la purificación rápida (60 s) y simultánea detección molecular de ADN de *B. pertussis* por qPCR o LAMP a partir de hisopados nasofaríngeos, con rendimiento muy comparable a la metodología convencional basada en columnas de sílice. El método rápido podría ser de mucha utilidad en el fortalecimiento del diagnóstico oportuno y vigilancia molecular de tos ferina en el primer nivel de atención que ayuden a controlar y prevenir la enfermedad.
